# Historical hydrometric context and explainable multi-horizon evaluation of a GloFAS-derived discharge product near Babahoyo, Ecuador

**DOI:** 10.3389/frai.2026.1901591

**Published:** 2026-08-21

**Authors:** Alexander Haro-Sarango, Silvia Cachay-Salcedo, Jessica Saavedra-Vasconez, Rosa Salcedo-Dávalos, Elizabeth Proaño-Altamirano

**Affiliations:** Escuela Profesional de Contabilidad, Universidad Peruana Unión, Lima, Peru

**Keywords:** Babahoyo, explainable machine learning, GloFAS, historical gauge context, INAMHI hydrological yearbooks, multi-horizon forecasting, temporal validation, uncertainty calibration

## Abstract

Open global hydrological products are often the only continuous source available for data-scarce locations, but their local use is stronger when the modelled signal is connected to documented hydrometric evidence. This study evaluates an explainable machine-learning workflow for anticipating the GloFAS v4-derived discharge series returned by the Open-Meteo Flood API near Babahoyo, Ecuador, and complements the modern 2017–2026 analysis with a historical hydrometric-context protocol based on official INAMHI yearbooks for the San Pablo-Babahoyo system. Direct models were assessed at t+1, t+3, t+7, and t+14 using leakage-aware features, rolling-origin backtesting, a 365-day holdout, prediction intervals, product-state classification, permutation importance, and SHAP. ElasticNetCV achieved R^2^/NSE of 0.900 at t+1 in the final holdout, while mean backtest skill weakened at longer horizons. The dominant role of current and recent discharge values shows that the main short-term contribution is efficient local post-processing of GloFAS persistence; meteorological and seasonal information becomes relatively more important as lead time increases. The study therefore contributes a transparent multi-horizon evaluation, an auditable pathway for historical gauge-level corroboration, and practical diagnostics for deciding when local emulation is informative and when the native global product should remain the primary reference.

## 1 Introduction

Prediction in ungauged basins is an established hydrological research field rather than a new problem created by this study. Regionalisation, conceptual rainfall-runoff models, satellite and reanalysis forcing, and regional machine-learning systems have all been evaluated for locations with limited or absent local observations, commonly by transferring information across basins and testing predictions against withheld gauge records (Hrachowitz et al., 2013; Kratzert et al., 2019). That observational comparison is essential: it distinguishes skill in representing a physical river from skill in reproducing another model product. The present analysis does not contain that comparison and is therefore positioned more narrowly as model-output emulation (Razavi et al., 2012).

Babahoyo, Ecuador, provides a relevant case for examining this narrower problem because the city occupies a low-relief, flood-prone setting influenced by river channels, drainage constraints, agricultural land, urban occupation, and hydraulic infrastructure. A continuous local discharge series suitable for this analysis was not obtained. This statement concerns data access for the study and should not be interpreted as evidence that hydrometric stations do not exist in the Guayas system. Establishing station identity, record completeness, and access conditions through the Instituto Nacional de Meteorología e Hidrología (INAMHI) remains necessary for physical validation.

The specific research opportunity lies in connecting three elements that are usually examined separately: a freely available global discharge product, a lightweight local and explainable forecasting layer, and historical hydrometric documentation for the study area. For Babahoyo, this combination makes it possible to quantify how much short-horizon regularity can be extracted from the GloFAS signal, determine which predictor families sustain that skill, evaluate calibration as lead time increases, and establish a reproducible route for comparison with historical INAMHI gauge levels. The novelty is therefore methodological integration and local diagnostic value, rather than a claim that prediction in ungauged basins is a new field.

The distinction is consequential because GloFAS is a global modelling system based on meteorological forcing, the LISFLOOD hydrological and routing model, and calibration or regionalisation informed by streamflow observations where available (Alfieri et al., 2013; Van der Knijff et al., 2010; Harrigan et al., 1979; Hirpa et al., 2018; Harrigan et al., 2023; Copernicus Emergency Management Service, 2025; Copernicus Emergency Management Service, 2024). Open-Meteo distributes reanalysis and forecast outputs from this product family and returns discharge for a selected grid cell rather than a local gauge measurement (Open-Meteo, 2026). Consequently, using Open-Meteo meteorology and lagged GloFAS-derived discharge to predict future GloFAS-derived discharge creates a dependent input-target system. High accuracy can demonstrate temporal regularity and emulatability of that product, but it cannot by itself establish accuracy for the Babahoyo River. Published global verification further shows that GloFAS skill varies substantially by location and lead time, including weak or negative longer-lead skill in parts of the western coast of South America (Harrigan et al., 2023).

The contribution of the study is a local post-processing and evaluation framework with three practical outputs. First, it provides transparent, horizon-specific evidence on the predictability of the GloFAS-derived signal. Second, it separates autoregressive memory from meteorological and seasonal contributions using explainability and input-family comparisons. Third, it defines an auditable historical-corroboration route using official INAMHI yearbooks and the Flood API at matching dates. Together, these elements offer a more informative basis for deciding when a local emulator adds analytical value and when direct use of the native product is preferable.

Accordingly, the objective is to evaluate the multi-horizon skill, calibration, interpretability, and local analytical value of explainable machine learning applied to a GloFAS v4-derived discharge product near Babahoyo at 1-, 3-, 7-, and 14-day horizons. A complementary objective is to establish a reproducible historical hydrometric-context procedure linking official INAMHI yearbooks for the San Pablo-Babahoyo system with matching Flood API dates. Transferability is treated as an empirical question for future multi-site replication rather than assumed from a single location.

## 2 Theoretical framework

The literature relevant to this study comprises three distinct tasks. The first is prediction in ungauged basins, where models are transferred or regionalised and ultimately evaluated against observed discharge withheld from model development (Hrachowitz et al., 2013; Kratzert et al., 2019). The second is large-scale hydrological forecasting, in which global or regional systems integrate meteorological forcing, hydrological process representation, routing, calibration, reanalysis, reforecasts, and real-time forecasts (Alfieri et al., 2013; Van der Knijff et al., 2010; Harrigan et al., 1979; Hirpa et al., 2018; Harrigan et al., 2023; Copernicus Emergency Management Service, 2025; Copernicus Emergency Management Service, 2024). The third is model emulation, where a statistical or machine-learning surrogate learns the behavior of a computational or externally produced target (Razavi et al., 2012). The present work belongs primarily to the third task because its target is GloFAS-derived rather than locally observed.

This classification changes the interpretation of performance. A model that predicts a model-derived series can be useful for compression, local explanation, sensitivity analysis, or rapid approximation, but its error is measured relative to the source product. Shared forcing, autoregressive target history, and common seasonality can produce high short-horizon scores without proving that the corresponding physical discharge is accurate. Published evaluations of ungauged forecasting and global flood systems therefore remain a stricter reference because they use gauge observations, hydrologically evaluated gridded inputs, reforecasts, or independent event evidence (Hrachowitz et al., 2013; Kratzert et al., 2019; Harrigan et al., 1979; Hirpa et al., 2018; Harrigan et al., 2023).

Evidence from Ecuador and the wider Andes reinforces the need for independent local validation. Comparisons of satellite and reanalysis temperature or precipitation products against station networks show spatially heterogeneous errors, sensitivity to topography and temporal aggregation, and improved hydrological performance when multisource fields are adjusted with gauge or streamflow information (Magoffin et al., 2023; Iza-Wong et al., 2026; Fernández-Palomino et al., 2022; Condom et al., 2020; Muñoz-Sabater et al., 2021). These studies do not validate the GloFAS cell used here, but they demonstrate why agreement with a global gridded product cannot substitute for verification against local observations or independent flood evidence.

A defensible emulation assessment requires four elements: comparison with persistence and with the native forecast product; time-ordered validation that prevents future information from entering preprocessing; uncertainty diagnostics that examine both coverage and interval width; and explanations that distinguish dependence on source-product memory from incremental meteorological information. These principles motivate the revised framing. Explainability is used to identify what the emulator relies on, not to infer causal river mechanisms, while the native GloFAS forecast is treated as the primary product benchmark rather than as an interchangeable alternative.

## 3 Materials and methods

The study adopted a quantitative, applied, non-experimental, retrospective longitudinal case-study design with a predictive product-emulation purpose. The analysis was fixed to latitude −1.80217 and longitude −79.53443, America/Guayaquil time, and to the daily period from 1 January 2017 through 28 March 2026; the end date is no longer defined relative to the date on which the code is executed. The merged base series contained 3,374 dates. After shifting the GloFAS-derived target, the regression samples were 3,373 observations for t+1, 3,371 for t+3, 3,367 for t+7, and 3,360 for t+14. These are samples from one coordinate and one evolving source product, not a multi-basin sample, so external transferability was not tested.

Three quantities are kept distinct throughout the revised manuscript: observed discharge means an in-situ gauge measurement, which is absent here; GloFAS-derived discharge means the gridded model output returned by the Flood API; and machine-learning forecast means an estimate produced by the local emulator with GloFAS-derived discharge as its target. The term gauge-free refers only to the absence of local gauge observations from this study workflow. It does not imply that GloFAS was developed independently of gauge information, because its global calibration and regionalisation use in-situ records where available (Hirpa et al., 2018; Copernicus Emergency Management Service, 2025; Copernicus Emergency Management Service, 2024).

The database integrated Open-Meteo Archive/Forecast meteorology with the Open-Meteo Flood API discharge product. Meteorological variables included maximum and minimum 2 m temperature, precipitation, rain, precipitation hours, maximum wind and gust speed, dominant wind direction, shortwave radiation, FAO reference evapotranspiration, and weather code. The Flood API returns simulated discharge from GloFAS at approximately 5 km resolution and cautions that the closest or largest represented river may not correspond exactly to the requested reach (Open-Meteo, 2026). The code scanned 25 nearby coordinate offsets for completeness and variability but retained the original coordinate manually. It did not verify the returned cell against a named Babahoyo/San Pablo reach, upstream drainage area, or channel identifier. The target is therefore described as a GloFAS-derived grid-cell signal near Babahoyo, not as a confirmed measurement or exact representation of a specific river reach.

The input-target dependence is methodologically central. GloFAS v4 uses LISFLOOD, meteorological forcing, globally calibrated parameters where observations are available, and parameter regionalisation elsewhere (Van der Knijff et al., 2010; Hirpa et al., 2018; Copernicus Emergency Management Service, 2025; Copernicus Emergency Management Service, 2024; Beck et al., 2016). The local emulator then uses meteorological variables and lagged values of that model-derived discharge to predict its future values. Errors in GloFAS, cell selection, forcing, routing, calibration, or regionalisation can therefore be learned and propagated. The evaluation quantifies agreement with the source product only; it cannot estimate bias against the physical river or adjudicate whether a native GloFAS forecast or an emulator forecast is closer to reality.

The evidential scope is a site-specific evaluation of local GloFAS post-processing. The analysis does not replace gauge-based hydraulic validation, but it now incorporates historical hydrometric documentation as an independent contextual layer and supplies a reproducible procedure for matching those records with Flood API dates. This strengthens the physical relevance of the case study without treating level and discharge as interchangeable variables.

### 3.1 Historical hydrometric-context and API-supported cell assessment

Official INAMHI hydrological yearbooks were incorporated as the documentary source for historical gauge information in the San Pablo-Babahoyo system, particularly station H371/H0371 San Pablo en Palmar (Instituto Nacional de Meteorología e Hidrología, 1990; Instituto Nacional de Meteorología e Hidrología, 1991; Instituto Nacional de Meteorología e Hidrología, 1992; Instituto Nacional de Meteorología e Hidrología, 1993; Instituto Nacional de Meteorología e Hidrología, 2026). The yearbook tables report daily water level rather than a directly comparable discharge series. Accordingly, the historical component is designed to evaluate temporal correspondence—seasonality, rank association, lagged association, timing of high-level episodes, and agreement in standardized anomalies—rather than unit-for-unit error between centimetres and cubic metres per second. This distinction permits meaningful external corroboration without requiring an unavailable rating curve.

The accompanying reproducible script downloads the official yearbooks, identifies pages containing H371/H0371, extracts or renders the relevant tables for verification, and queries the Open-Meteo Flood API for matching historical dates. Candidate GloFAS cells around the station and the original Babahoyo coordinate are compared using completeness, Spearman and Kendall association, cross-correlation across ±10-day lags, monthly-cycle similarity, and high-state event agreement. Cell selection is therefore based on correspondence with independent historical hydrometric behavior rather than on variance alone. Where automated extraction is uncertain, the script flags the affected table for manual verification instead of silently accepting OCR output.

This two-period design keeps inference disciplined. Historical INAMHI levels provide local hydrometric context and a basis for cell screening, whereas the 2017–2026 period supports the modern forecasting experiments. The historical evidence is not used to claim direct accuracy for 2026; it is used to improve geographic and hydrological justification for the selected global-product signal.

### 3.2 Data preparation, feature engineering, and target construction

Feature engineering used only information available at or before forecast origin t. It included calendar and Fourier seasonality terms, thermal range, a water-balance proxy, rainfall intensity, gust factor, antecedent precipitation indices, wet/dry spells, rainfall thresholds, backward-looking precipitation and discharge windows, lags, changes, anomalies, and interactions. Future targets were created only after these predictors. The merged daily calendar had no systematic missing dates; however, lag, difference, and rolling operations necessarily generated leading missing feature values. This resolves the apparent contradiction in the original text: absence of calendar gaps does not mean absence of engineered missing values. Fully empty columns were removed, and remaining feature missingness was median-imputed within each training partition only.

Regression targets were future GloFAS-derived discharge at t+1, t+3, t+7, and t+14. The binary high-product-state target was defined as next-day GloFAS-derived discharge at or above its 0.90 quantile; multicategory states were Normal (<0.80), Watch (0.80- < 0.90), Warning (0.90- < 0.97), and Extreme (≥0.97). In the archived run, these quantiles were calculated from the full series before the temporal split. This introduces mild label-definition leakage. The classification results are therefore retained only as exploratory sensitivity analyses and are not used to support the core regression conclusions. A fully leakage-free rerun must estimate all cutoffs within each training partition and then apply the fixed training cutoffs to validation and test data.

Four feature families were implemented in the analysis code: core combined inputs, weather-only inputs, discharge-history-only inputs, and an augmented sensitivity set containing same-day Flood API descriptors. The submitted repository does not archive the generated split-level ablation tables, so the incremental skill of meteorology beyond discharge history cannot be independently verified from the available materials. No revised claim is therefore made that meteorological predictors add predictive value beyond autoregressive GloFAS memory. The persistence baseline and variable-importance results are interpreted as evidence that the t+1 emulator is principally source-product autoregression.

### 3.3 Regression models, baselines, and temporal validation

Regression benchmarks were one-step persistence and seven-day seasonal persistence. Candidate models were ElasticNetCV, ExtraTreesRegressor, HistGradientBoostingRegressor, and XGBoost. The archived fast configuration used random seed 42; ElasticNetCV tested l1 ratios 0.1, 0.5, 0.9, and 1.0 with threefold internal cross-validation; ExtraTrees used 250 trees, minimum leaf size 2, and max_features = 0.65; HistGradientBoosting used learning rate 0.03, maximum depth 6, 250 iterations, minimum leaf size 20, and L2 regularisation 0.1; XGBoost used 250 trees, depth 5, learning rate 0.04, subsample and column subsample 0.85, and reg_lambda = 1.0. These were fixed configurations rather than an exhaustive, nested hyperparameter search. Consequently, the results do not establish a general superiority of linear over tree-based models.

Validation used an expanding-window rolling origin with three non-overlapping 90-day test blocks, preserving temporal order rather than randomly shuffling time-series observations (Bergmeir and Benítez, 2012). Split 1 trained on 1 January 2017–31 December 2019 and tested forecast origins from 1 January-30 March 2020; Split 2 trained through 30 March 2020 and tested 31 March-28 June 2020; Split 3 trained through 28 June 2020 and tested 29 June-26 September 2020. These 270 test origins cover only part of 2020 and do not provide a complete representation of all wet seasons, dry seasons, or high-flow episodes in the nine-year record. The final holdout contained 365 common target dates from 29 March 2025 through 28 March 2026. The corresponding origin windows were 28 March 2025–27 March 2026 for t+1, 26 March 2025–25 March 2026 for t+3, 22 March 2025–21 March 2026 for t+7, and 15 March 2025–14 March 2026 for t+14. Holdout models were retrained on all earlier origin dates, and no holdout date overlapped the 2020 backtest blocks. R^2^ was calculated with the evaluation-period mean in the denominator; under that definition it is numerically identical to Nash-Sutcliffe efficiency (NSE), whose zero benchmark corresponds to the evaluation-period mean (Nash and Sutcliffe, 1970; Knoben et al., 2019). Only single-seed point estimates and split averages were retained in the manuscript; no repeated-run dispersion or Diebold-Mariano comparison of predictive accuracy was available, so model rankings are descriptive rather than inferential (Diebold and Mariano, 1995).

### 3.4 Uncertainty, exploratory classification, prospective forecasting, and explainability

Diagnostic 80% intervals were estimated by fitting separate HistGradientBoosting quantile models at 0.10, 0.50, and 0.90, following the general quantile-regression principle of estimating conditional distributional points rather than only the conditional mean (Koenker and Bassett, 1978). The archived implementation evaluated t+1, t+7, and t+14 but inadvertently omitted t+3; Table 1 now reports that omission explicitly rather than implying complete horizon coverage. No non-crossing constraint or monotone rearrangement was imposed on the independently fitted quantiles, although such procedures are specifically designed to correct quantile crossing (Chernozhukov et al., 2010). Interval coverage and width are therefore interpreted as diagnostic properties of the archived run, not as a fully calibrated probabilistic forecasting system.

**Table 1:** Diagnostic 80% prediction-interval coverage and width for horizons implemented in the archived run. The missing t+3 analysis is shown explicitly.

Horizon (days)	Empirical 80% interval coverage	Average interval width (m^3^/s)
1	0.745	81.221
7	0.597	164.952
14	0.548	135.189

The exploratory binary module tested logistic regression, ExtraTreesClassifier, HistGradientBoostingClassifier, and XGBoostClassifier. A final 120-day, time-ordered validation segment within the training data was used to select a probability cutoff by maximising F₂, where recall receives four times the weight of precision. The model was then refitted on all pre-holdout data and evaluated once on the holdout. Accuracy, balanced accuracy, precision, recall, F1, receiver-operating-characteristic area under the curve (ROC-AUC), precision-recall area under the curve (PR-AUC), and Brier score were reported. Because the class labels used full-series quantiles, these metrics are exploratory.

The multicategory module used the same exploratory state definitions and compared ExtraTreesClassifier with HistGradientBoostingClassifier using accuracy, balanced accuracy, Macro-F1, and Weighted-F1. These are classifications of GloFAS-derived product states, not observed flood severity categories. The label names Normal, Watch, Warning, and Extreme are communication labels based on empirical product quantiles and are not official hydrological or civil-defence thresholds.

Two prospective outputs were generated after fitting on the available history: direct estimates at the four lead times and a recursive daily path from the t+1 emulator. They were compared with the contemporaneous native Flood API forecast only as a single-issuance consistency check. Historical native forecasts at matching issuance dates were not archived, so this comparison is not a backtest and cannot establish incremental skill over GloFAS. Because operational GloFAS and the Flood API already supply forecasts at these horizons (Harrigan et al., 2023; Open-Meteo, 2026), added value would require a matched historical comparison against archived native forecasts and, ultimately, observed discharge.

Permutation importance was calculated for t+1, t+7, and t+14, and SHAP was applied to the t+1 emulator. These methods identify predictive dependence within the fitted product-emulation model. They do not establish causal hydrological mechanisms, particularly because the dominant predictor is the current value of the same GloFAS-derived series used to define the future target.

## 4 Results

### 4.1 Historical hydroclimatic behavior

**Figure 1:**
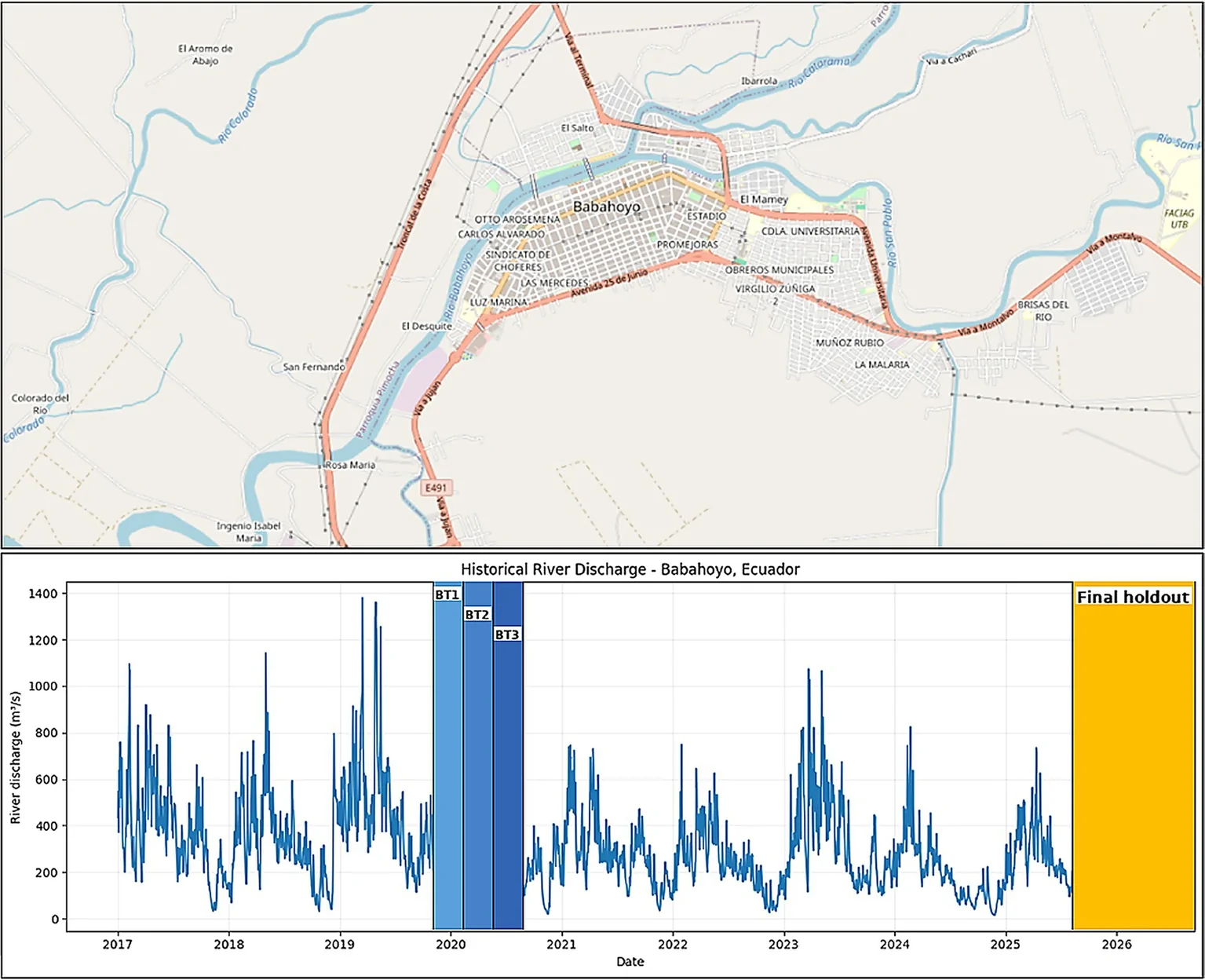
Territorial context and GloFAS-derived discharge series near Babahoyo, Ecuador. The upper panel presents the map context; the lower panel presents the 2017–2026 model-derived discharge signal. Shaded bands identify the three rolling-origin test blocks (BT1-BT3) and the final holdout target period. The map does not verify the identity of the GloFAS river cell or constitute gauge validation. ©OpenStreetMap contributors’. Source: OpenStreetMap contributors; License: Open Database License (ODbL). The map was generated/adapted by the authors; analytical overlays and lower time-series panel are original.

The modern series covered 1 January 2017–28 March 2026 and represents a GloFAS/Open-Meteo model-derived grid-cell signal near Babahoyo. It displayed a clear seasonal cycle, sustained hydrological memory, episodic high-value periods, and prolonged recessions, providing a structured signal for evaluating local multi-horizon post-processing. Official INAMHI hydrological yearbooks were also identified as the historical documentary source for H371/H0371 San Pablo en Palmar. Their inclusion establishes a concrete pathway for evaluating local seasonality and high-level timing against matching historical Flood API dates, while respecting the difference between observed level and modelled discharge (see Figure 1).

**Figure 2:**
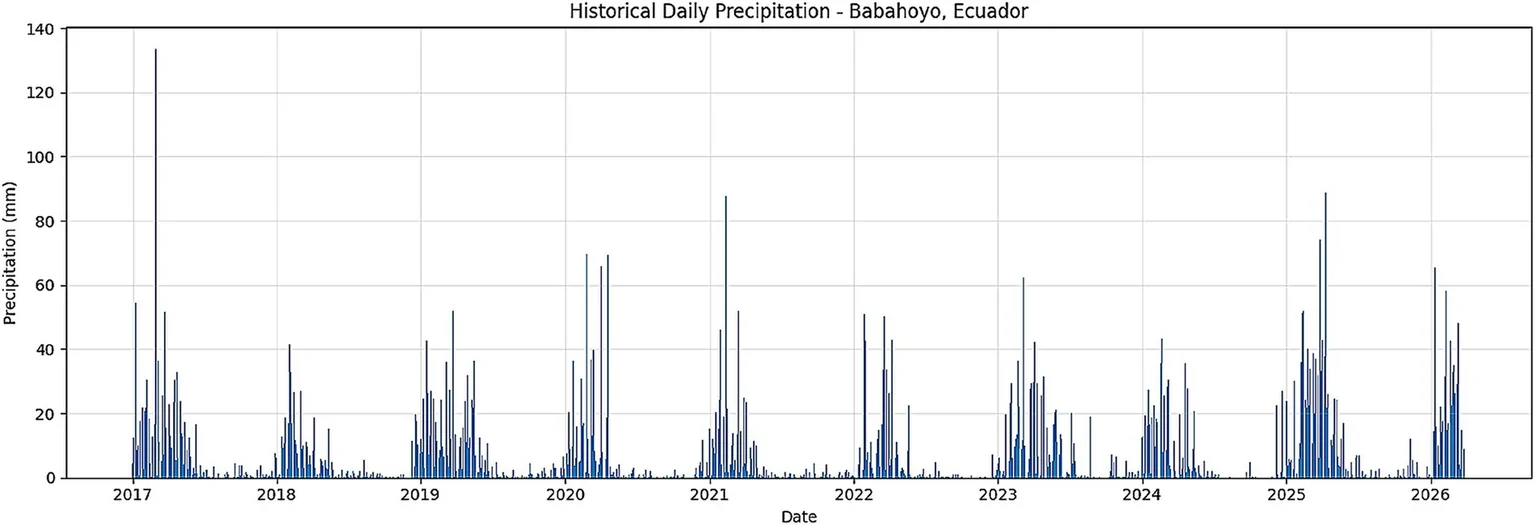
Historical daily Open-Meteo precipitation at the Babahoyo study coordinate from 1 January 2017 to 28 March 2026.

Open-Meteo precipitation was intermittent, with many low- or zero-rainfall days and fewer intense events. Babahoyo lies in a low alluvial setting connected to the wider Guayas system and surrounded by agricultural and urban land uses. These territorial characteristics provide context for why local process information could matter, but they were not available as continuous predictors and cannot be used to validate the timing or magnitude of peaks in the GloFAS-derived series (see Figure 2).

### 4.2 GloFAS-product emulation performance in backtesting and final holdout

Across the three 90-day backtest blocks, average emulation performance declined as the horizon increased. ElasticNetCV produced the highest mean R^2^ within the reported core set: 0.777 at t+1, 0.334 at t+3, −0.065 at t+7, and −0.609 at t+14. These values show that short-term regularity in the GloFAS-derived target is emulatable, whereas the archived local model loses mean skill relative to the evaluation-period mean at longer leads. Because all three blocks fall within January–September 2020, the gradient is a limited temporal diagnostic rather than a robust estimate across the full range of years and hydrological conditions (see Table 2).

**Table 2:** Mean performance across three non-overlapping 90-day rolling-origin test blocks for emulating future GloFAS-derived discharge, with the persistence baseline.

Horizon (days)	Best model	Best model R^2^/NSE	RMSE (m^3^/s)	MAPE (%)	Persistence t − 1 baseline R^2^/NSE
1	ElasticNetCV	0.777	62.832	13.965	0.430
3	ElasticNetCV	0.334	108.685	29.671	0.109
7	ElasticNetCV	−0.065	134.283	41.064	−0.240
14	ElasticNetCV	−0.609	158.869	51.532	−0.763

Split-specific dispersion was not retained in the submitted artifact.

**Figure 3:**
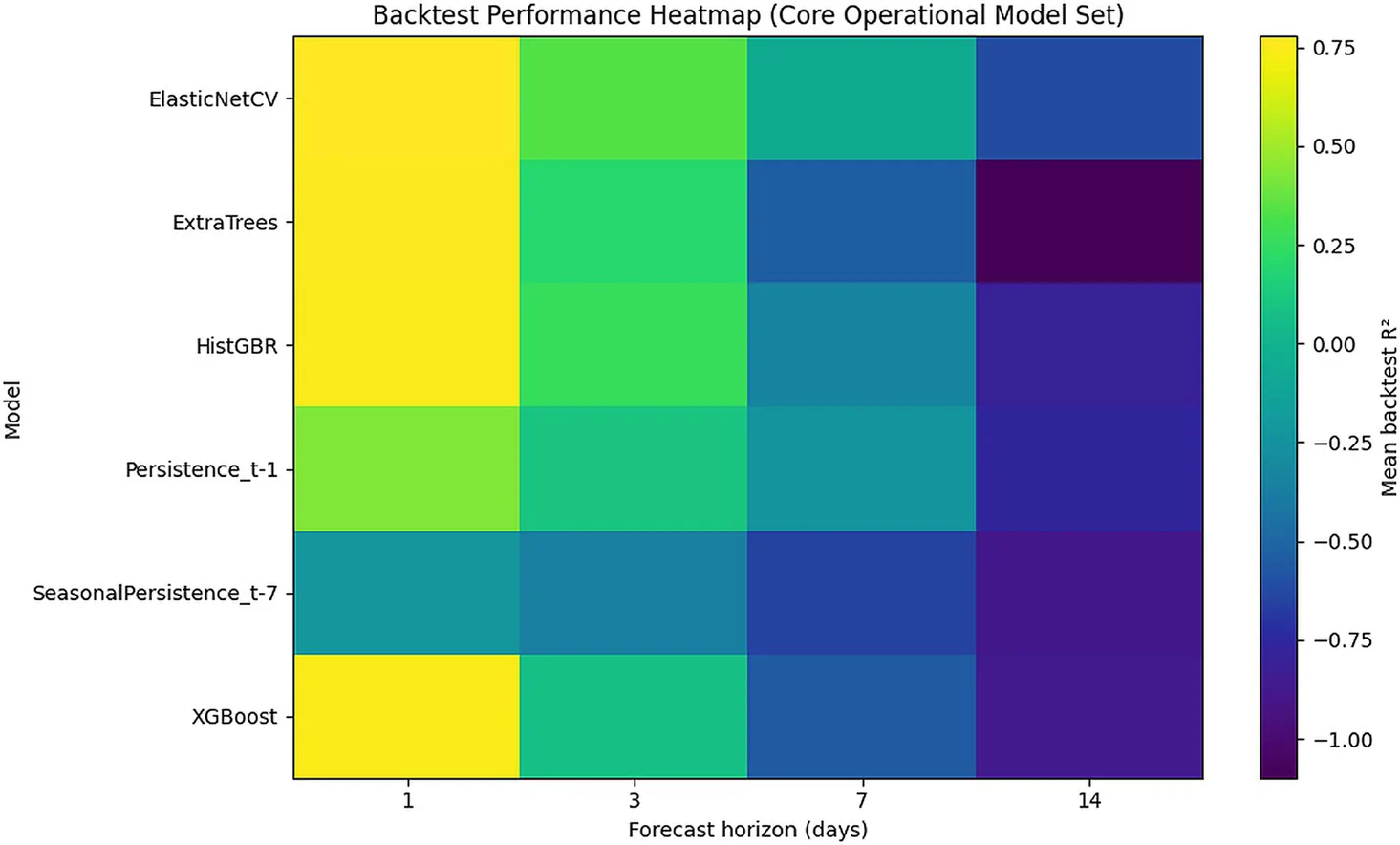
Mean rolling-origin R^2^ for the core product-emulation models at t+1, t+3, t+7, and t+14. Values are averages across three 2020 test blocks.

Figure 3 visualises the horizon gradient. The strongest result is the substantial improvement over persistence at t+1, demonstrating that the local feature set and regularised model capture more than a naive carry-forward rule. At t+3 the model retains positive average skill, while t+7 and t+14 identify the horizon at which the current local configuration requires stronger meteorological forcing, broader temporal sampling, or direct reliance on the native GloFAS forecast. The model comparison is descriptive because all candidates used fixed configurations and one random seed.

The final holdout produced stronger agreement with the GloFAS-derived target than the backtest average: R^2^/NSE was 0.900 at t+1, 0.687 at t+3, 0.535 at t+7, and 0.424 at t+14. These values apply to common target dates from 29 March 2025 to 28 March 2026 and do not validate the physical river. Agreement weakened with lead time through increasing RMSE, peak smoothing, timing differences, and reduced reproduction of abrupt changes.

The difference between the t+14 mean backtest result (−0.609) and final-holdout result (0.424) is scientifically informative: the emulator is sensitive to hydrological regime and evaluation period. Rather than invalidating the approach, this result identifies temporal stability as the principal target for improvement. More rolling origins distributed across wet and dry seasons, split-specific uncertainty, and matched native-GloFAS forecasts would determine when the local model is beneficial and when the global benchmark should be preferred (see Table 3).

**Table 3:** ElasticNetCV agreement with the GloFAS-derived target in the final 365-target-day holdout (29 March 2025–28 March 2026).

Horizon (days)	MAE (m^3^/s)	RMSE (m^3^/s)	MAPE (%)	R^2^/NSE
1	28.035	43.911	12.500	0.900
3	54.560	77.456	27.641	0.687
7	67.807	94.419	36.556	0.535
14	74.727	105.171	38.204	0.424

Forecast-origin windows differ by horizon as specified in Section 3.

**Figure 4:**
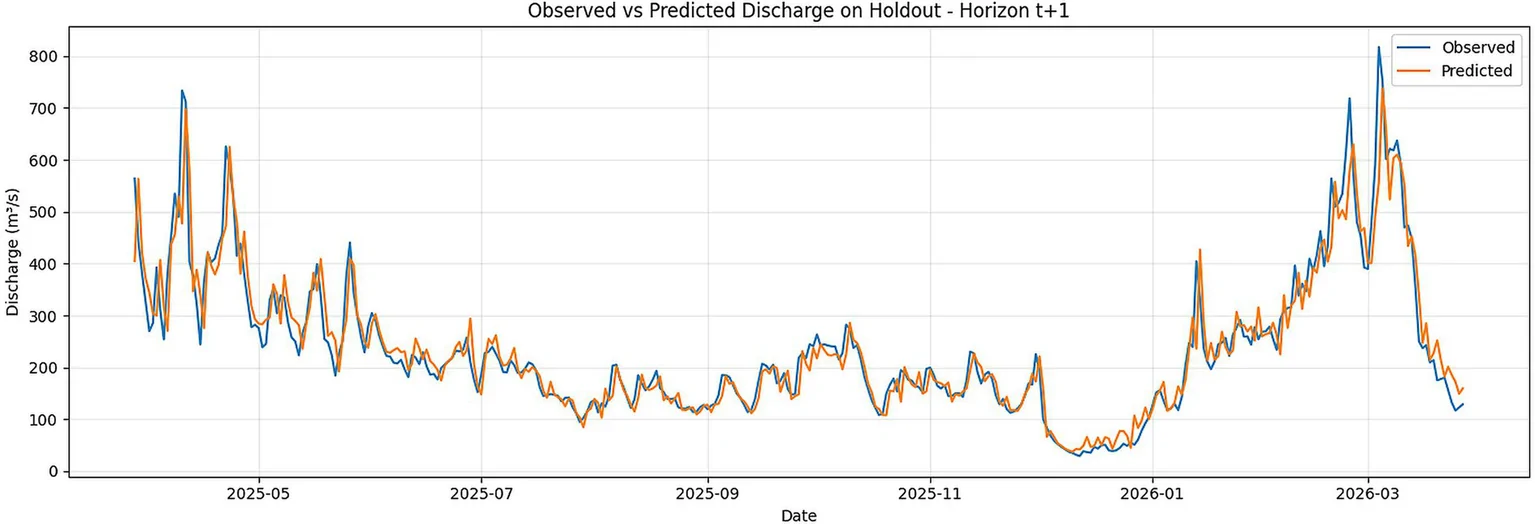
GloFAS-derived reference versus emulator prediction in the final holdout at t+1. The embedded legend label “observed” denotes the model-derived reference product, not gauge data.

**Figure 5:**
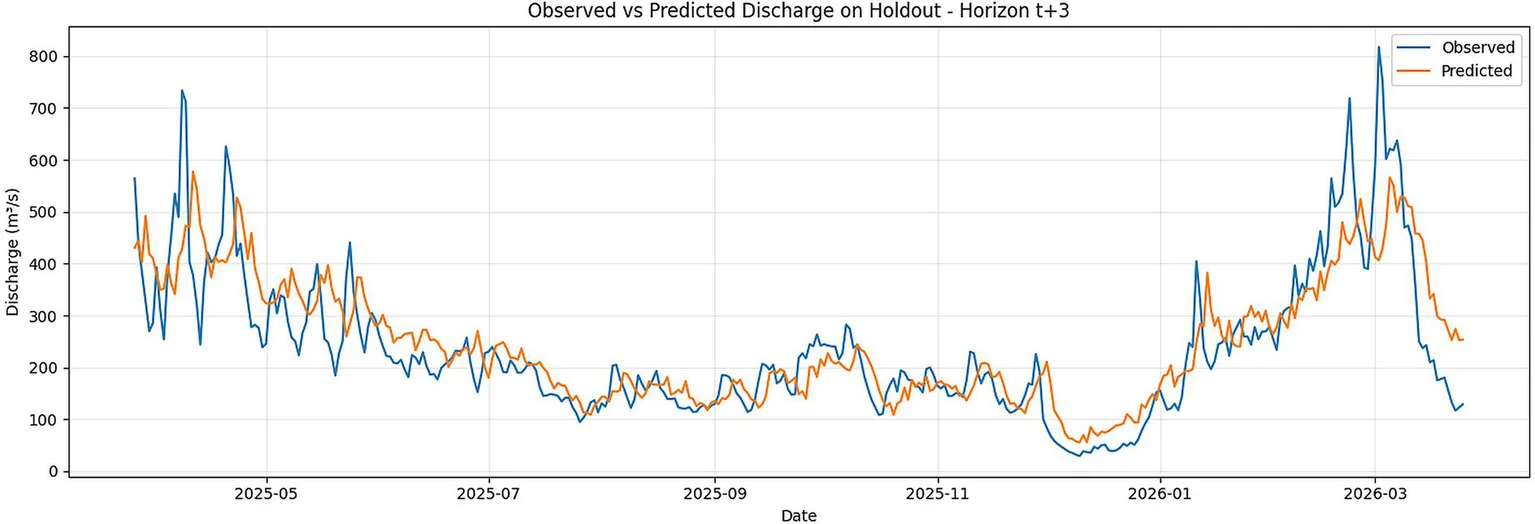
GloFAS-derived reference versus emulator prediction in the final holdout at t+3. The embedded legend label “observed” denotes the model-derived reference product.

**Figure 6:**
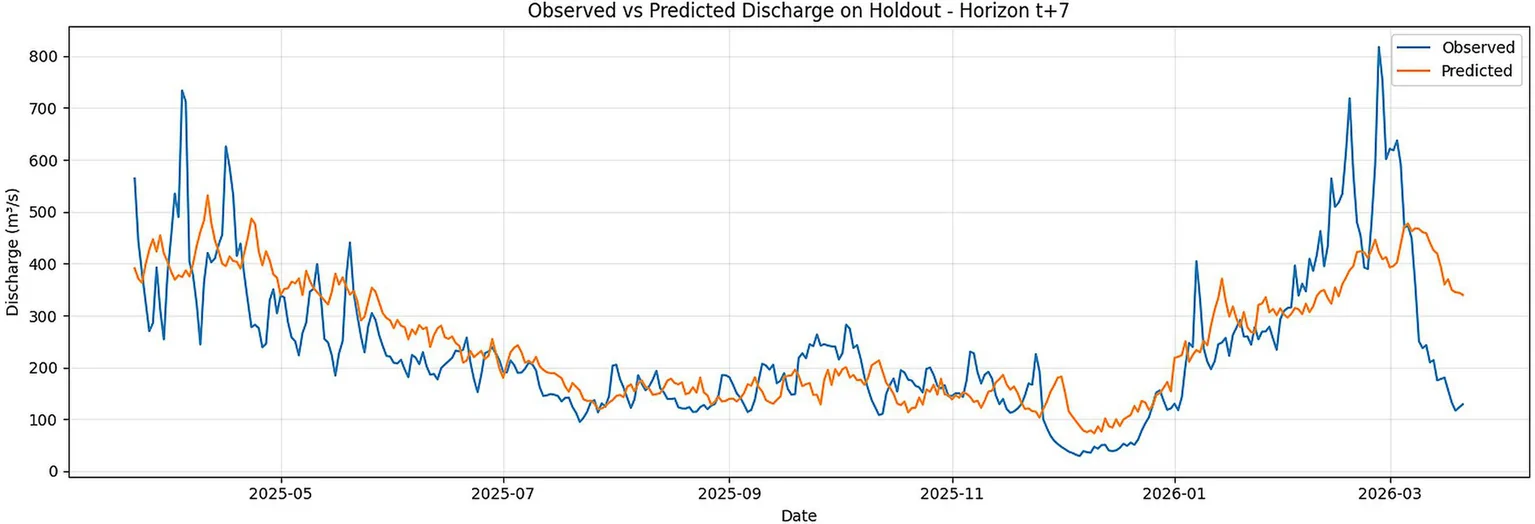
GloFAS-derived reference versus emulator prediction in the final holdout at t+7. The embedded legend label “observed” denotes the model-derived reference product.

**Figure 7:**
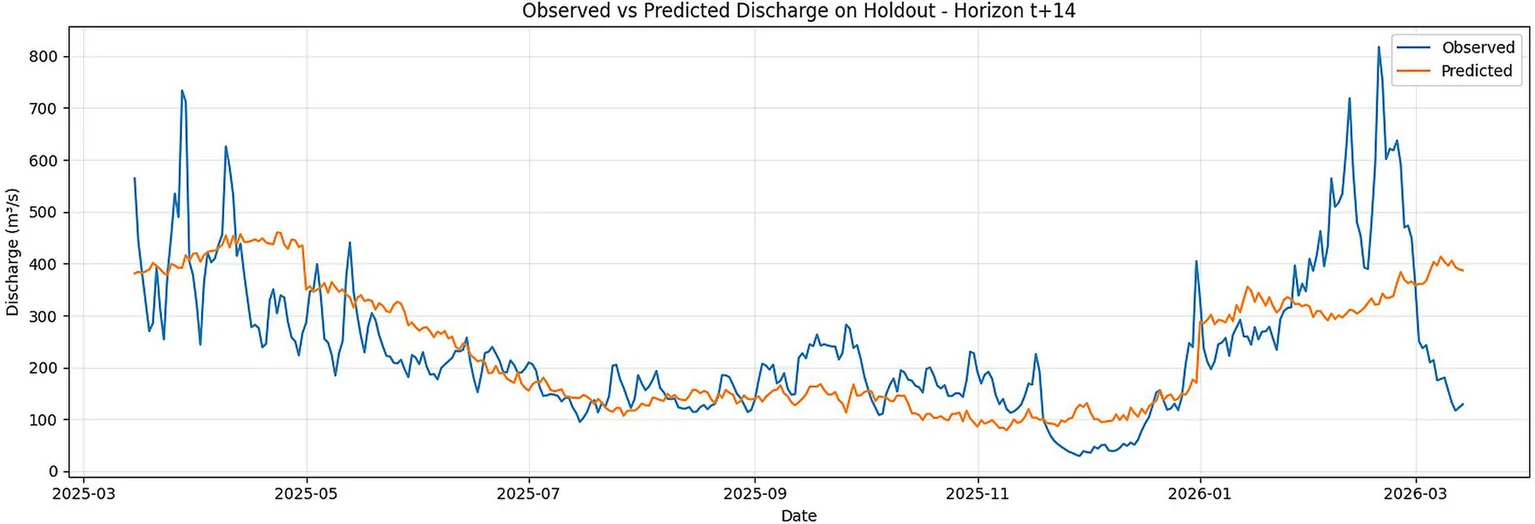
GloFAS-derived reference versus emulator prediction in the final holdout at t+14. The embedded legend label “observed” denotes the model-derived reference product.

Figures 4–7 compare the GloFAS-derived reference series with emulator predictions. In the embedded software figures, the legend term “Observed” refers to the GloFAS-derived target and not to a hydrometric gauge observation. Agreement is closest at t+1, while t+3 introduces timing and amplitude differences and t+7/t+14 increasingly smooth high values and abrupt transitions.

### 4.3 Diagnostic prediction intervals and residual behavior for the GloFAS-derived target

Empirical coverage of the nominal 80% interval was 74.5% at t+1, 59.7% at t+7, and 54.8% at t+14. Average width increased from 81.221 m^3^/s at t+1 to 164.952 m^3^/s at t+7, but then narrowed to 135.189 m^3^/s at t+14 while coverage deteriorated further. This non-monotonic width pattern is important: the t+14 problem is not simply greater uncertainty producing wider bands; it indicates mis-centred or under-dispersed intervals that fail to bracket the source-product target despite a still-large width. The t+3 interval was not estimated in the archived run.

**Figure 8:**
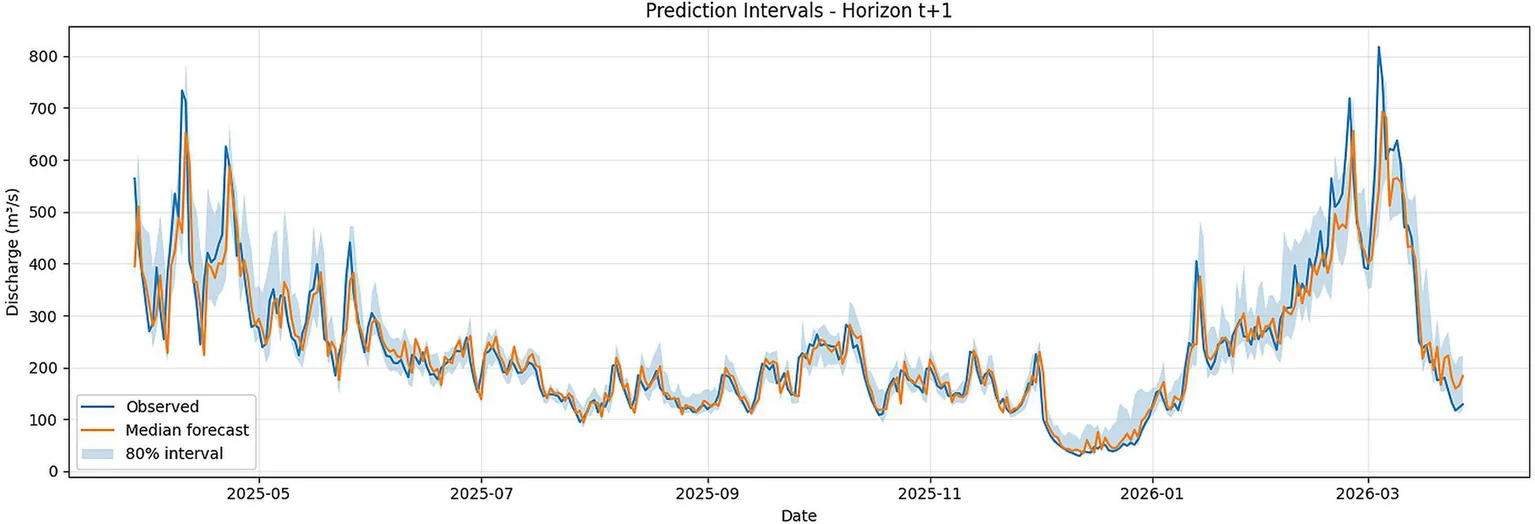
Diagnostic 80% interval for emulating the GloFAS-derived target at t+1.

**Figure 9:**
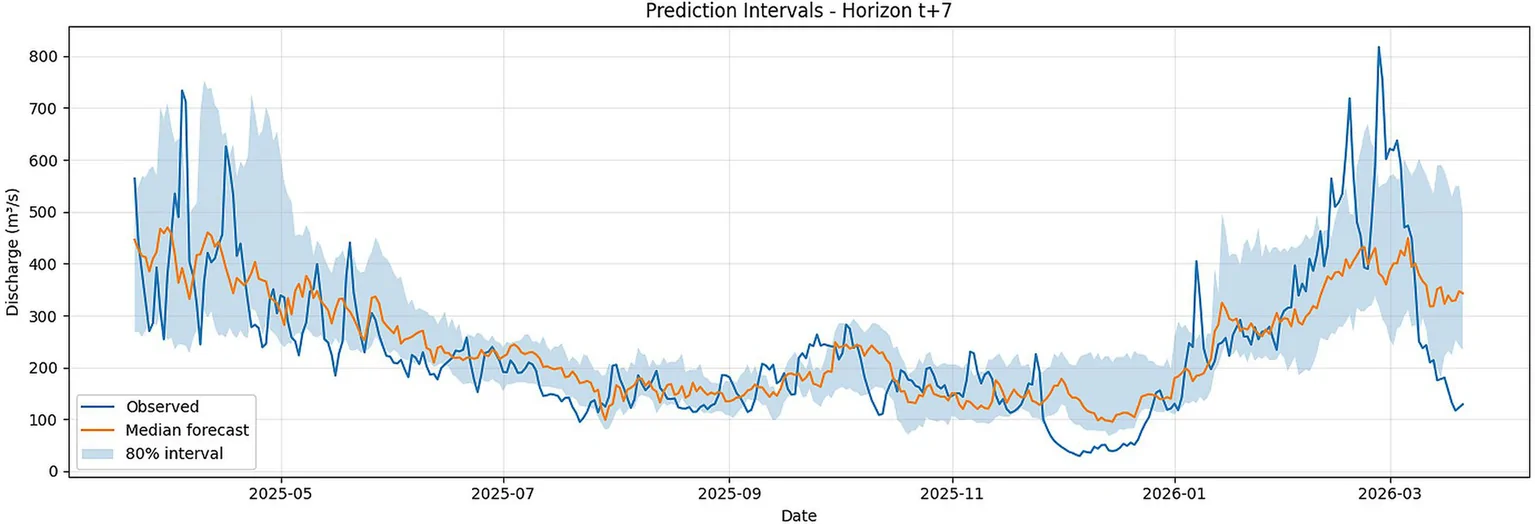
Diagnostic 80% interval for emulating the GloFAS-derived target at t+7.

**Figure 10:**
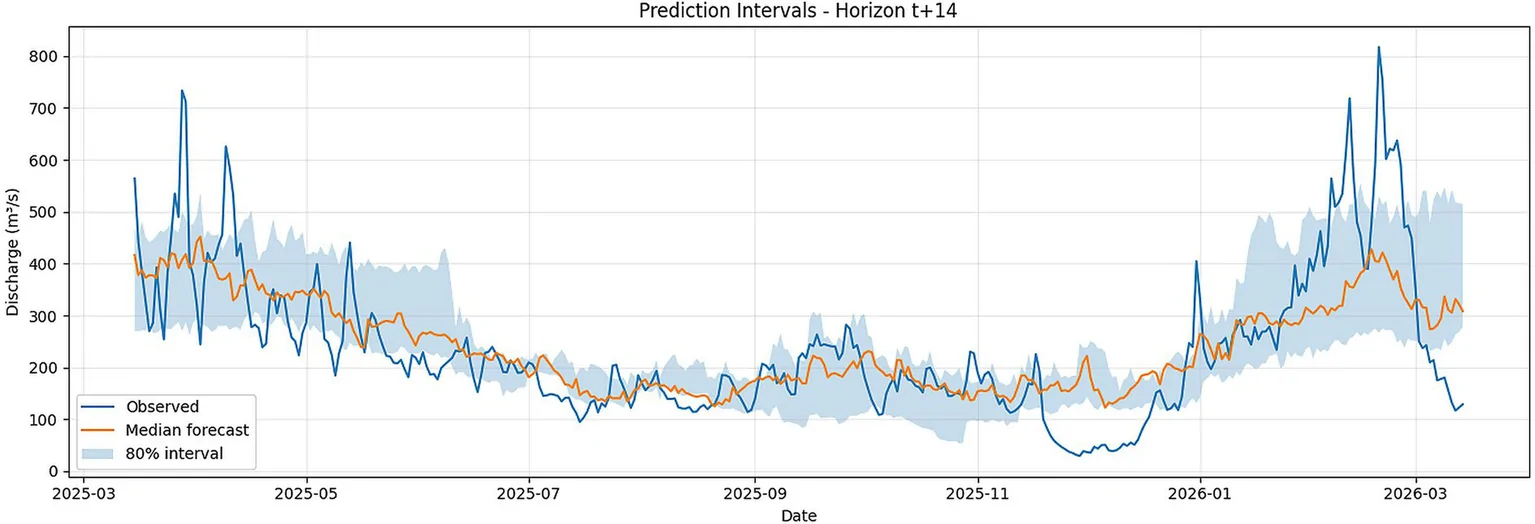
Diagnostic 80% interval for emulating the GloFAS-derived target at t+14.

Figures 8–10 confirm undercoverage, particularly around high values. As with the point-forecast plots, “Observed” denotes the GloFAS-derived reference. Because the quantiles were fitted independently without a non-crossing constraint or monotone rearrangement (Chernozhukov et al., 2010), and because no gauge observations were available, these intervals quantify uncertainty around product emulation only.

**Figure 11:**
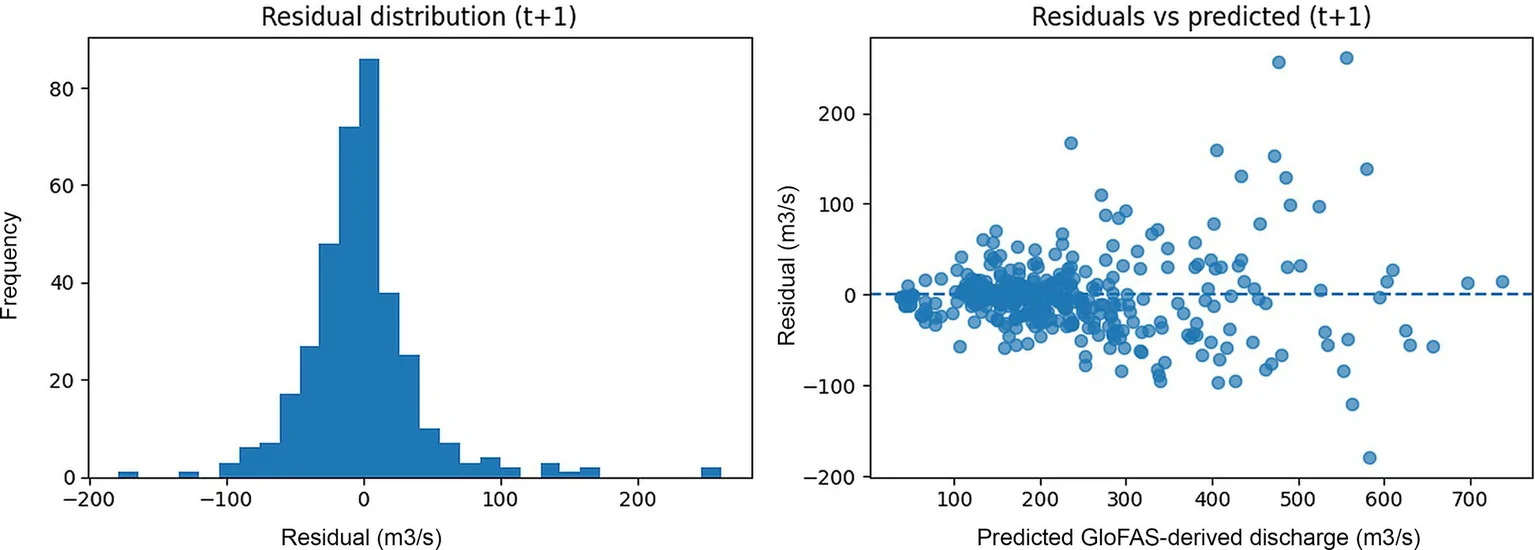
Residual diagnostics for the t+1 product-emulation model: residual distribution and residuals versus predicted GloFAS-derived discharge. The defective Q-Q panel was removed.

Residual diagnostics at t+1 showed a distribution centred near zero but with long tails and increasing dispersion at larger predicted values. The original Q-Q panel used an inappropriate scale that compressed theoretical quantiles into a near-vertical line; it has been removed rather than interpreted. The retained histogram and residual-versus-prediction plot support only the limited conclusion of heteroscedastic error relative to the GloFAS-derived target (see Figure 11).

### 4.4 Input-family dependence, variable importance, and model explainability

At t+1, contemporaneous GloFAS-derived discharge dominated permutation importance (0.6969), followed far behind by the three-day minimum of the same product (0.0363) and precipitation hours (0.0212). This hierarchy indicates that short-horizon performance is principally autoregressive emulation of source-product memory. It should not be interpreted as independent learning of the physical rainfall-runoff response.

At t+7 and t+14, importance shifted toward temperature, calendar, wet-season, and accumulated-rainfall terms, but their absolute importance was much smaller than the t+1 source-discharge contribution. The shift is consistent with the fading of direct autoregressive information and increasing reliance on coarse seasonality. Because the quantitative weather-only and discharge-history-only outputs were not archived with the submission, the incremental contribution of meteorology cannot be stated numerically (see Table 4).

**Table 4:** Leading predictors of the GloFAS-product emulator according to permutation importance.

Horizon	t+1	t+7	t+14
Predictor 1	river_discharge	temperature_2m_min	is_wet_season_ec
Importance	0.6969	0.0093	0.0951
Predictor 2	flow_min_3d	annual_sin_3	annual_cos_3
Importance	0.0363	0.0060	0.0334
Predictor 3	precipitation_hours	year	heavy25_count_60d
Importance	0.0212	0.0025	0.0246
Predictor 4	radiation_rain_interaction	heavy50_count_14d	annual_sin_3
Importance	0.0042	0.0019	0.0132
Predictor 5	flow_pctchg_1	gust_factor	year
Importance	0.0019	0.0015	0.0082

These values describe model dependence and are not estimates of causal hydrological effects or incremental skill.

**Figure 12:**
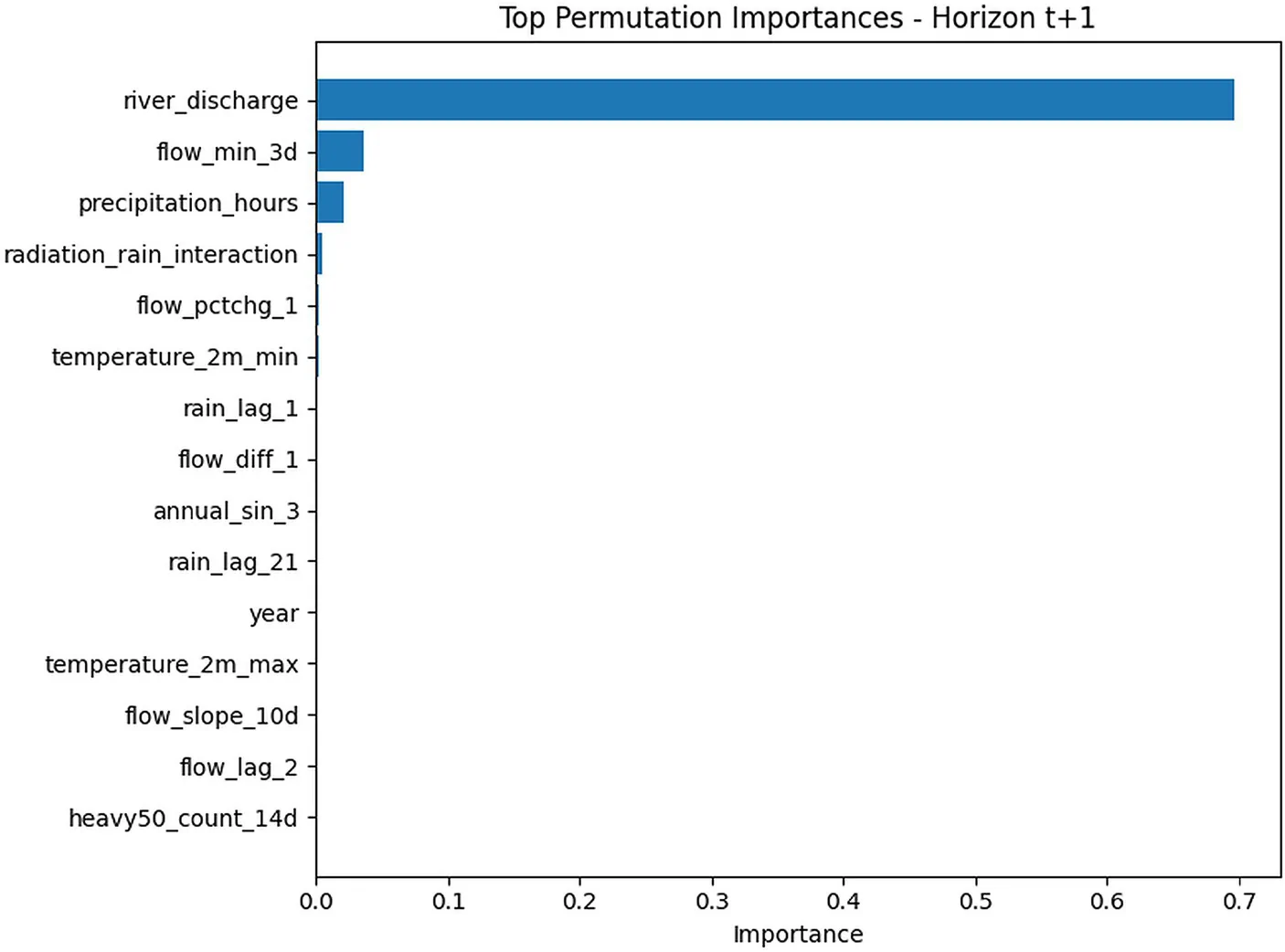
Permutation importance of the leading predictors for the t+1 horizon.

**Figure 13:**
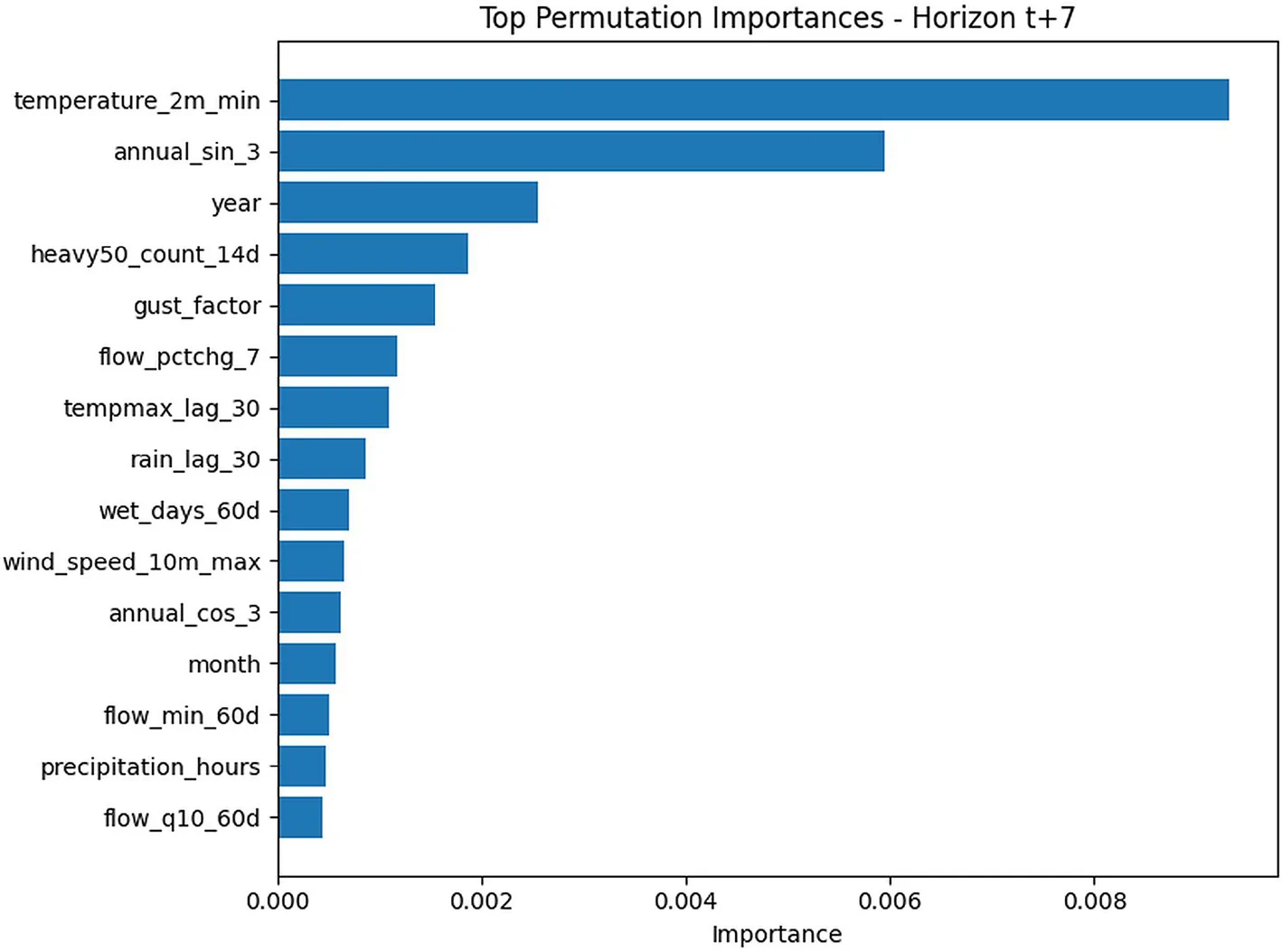
Permutation importance of the leading predictors for the t+7 horizon.

**Figure 14:**
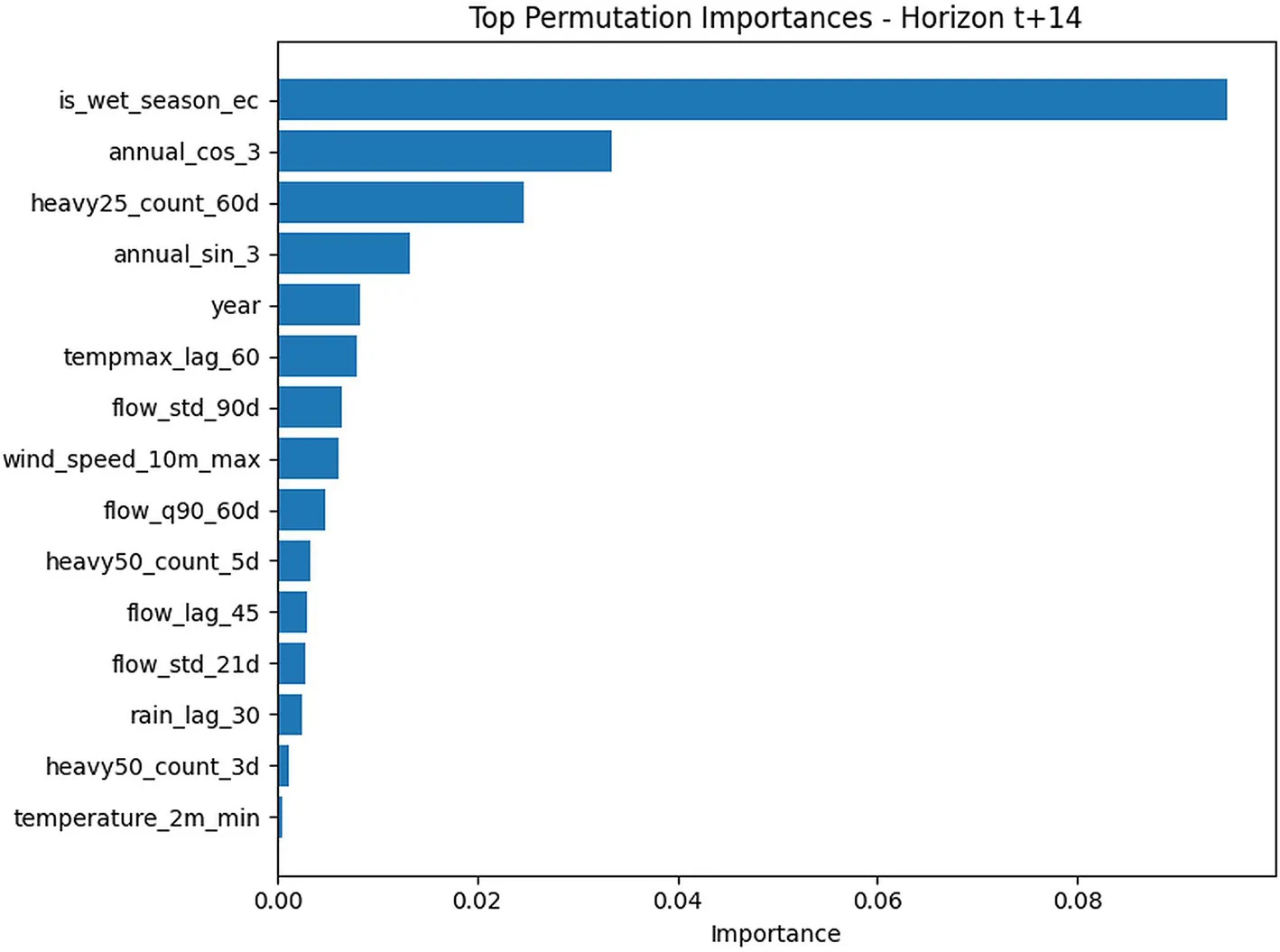
Permutation importance of the leading predictors for the t+14 horizon.

Figures 12–14 illustrate the change in dependence across horizons. The overwhelming t+1 contribution of the current GloFAS-derived value reinforces the interpretation of short-term autoregressive emulation. At longer horizons, the model falls back on broad climatic timing variables rather than verified local hydraulic processes.

**Figure 15:**
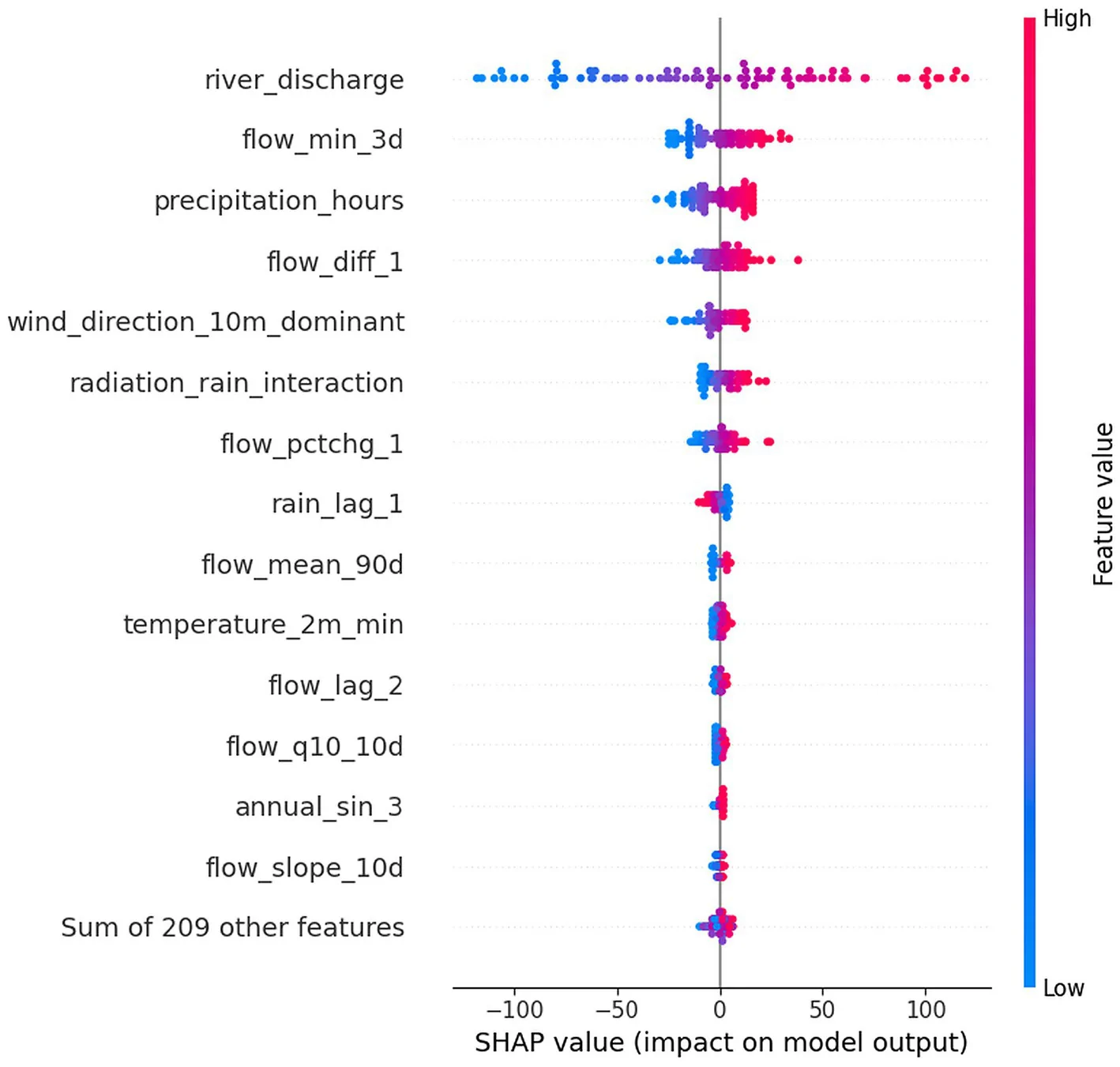
SHAP summary for the t+1 GloFAS-product emulator. Contributions describe dependence within the fitted model, not causal river mechanisms.

**Figure 16:**
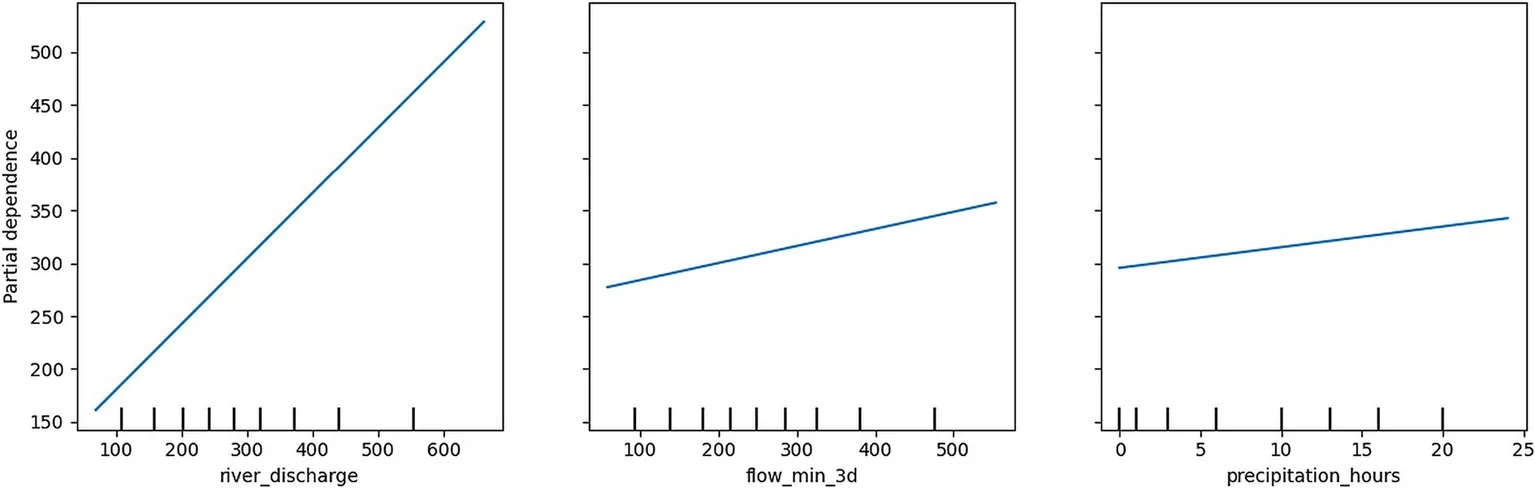
Partial dependence of the t+1 emulator on current GloFAS-derived discharge, three-day minimum product discharge, and precipitation hours.

SHAP and partial-dependence outputs show that higher current GloFAS-derived discharge, recent minimum product values, and precipitation hours are associated with higher emulator predictions. These are associational explanations within a dependent model-product system. They do not establish an operational-causal architecture for the Babahoyo River (see Figures 15, 16).

### 4.5 Prospective consistency check against the native flood API forecast

Direct emulator outputs were 156.422 m^3^/s at t+1, 239.907 m^3^/s at t+3, 320.742 m^3^/s at t+7, and 388.270 m^3^/s at t+14. These are single-issuance product-emulation scenarios fitted on the available history. They are not validated river forecasts and should not be interpreted as adding value over the native GloFAS forecast (see Table 5).

**Table 5:** Single-issuance direct forecasts from the GloFAS-product emulator.

Horizon (days)	Forecast date	Predicted discharge (m^3^/s)	Model
1	2026-03-29	156.422	ElasticNetCV
3	2026-03-31	239.907	ElasticNetCV
7	2026-04-04	320.742	ElasticNetCV
14	2026-04-11	388.270	ElasticNetCV

The values are scenarios, not independently validated river-discharge forecasts.

**Figure 17:**
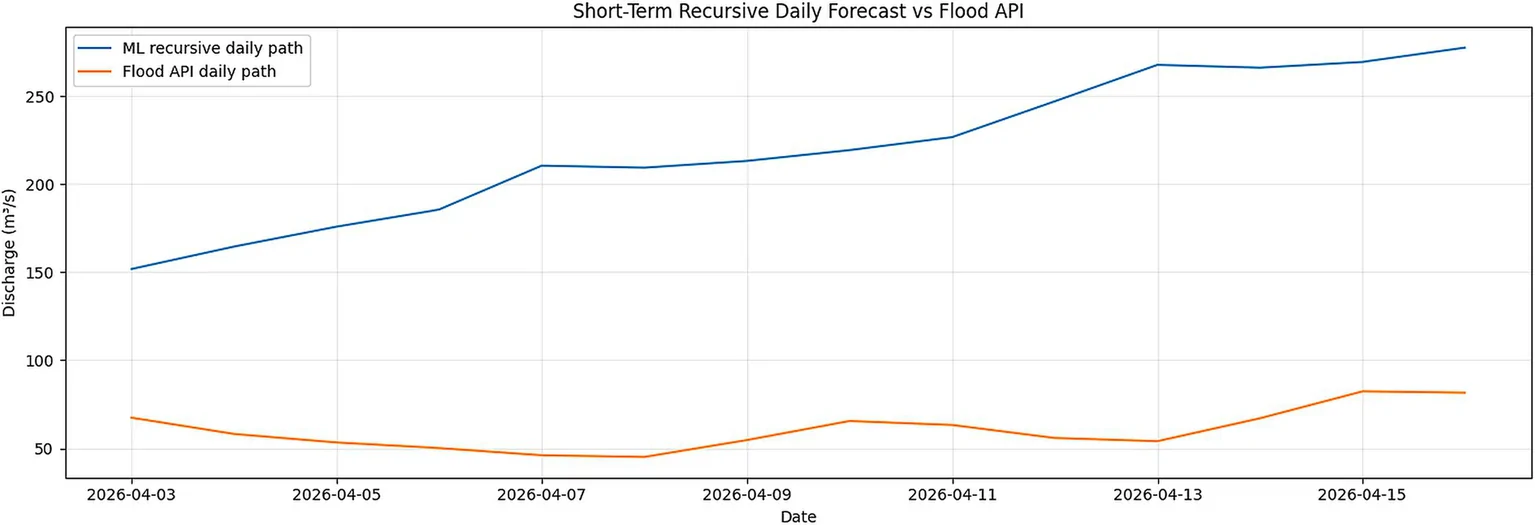
Single-issuance comparison of the recursive emulator path with the native Flood API trajectory. The divergence is a consistency failure that cannot be adjudicated without independent observations.

The recursive t+1 path rose from 151.806 to 277.372 m^3^/s, whereas the native Flood API path over matched dates was approximately 45–82 m^3^/s, producing a mean difference near 160 m^3^/s. This divergence is not evidence of two equally plausible open-data views. It shows that the recursive emulator drifted materially away from the product family used to define its historical target. Without a local gauge or archived native forecasts for historical issuance dates, the discrepancy cannot be resolved and weakens any claim of prospective usefulness (see Figure 17).

**Figure 18:**
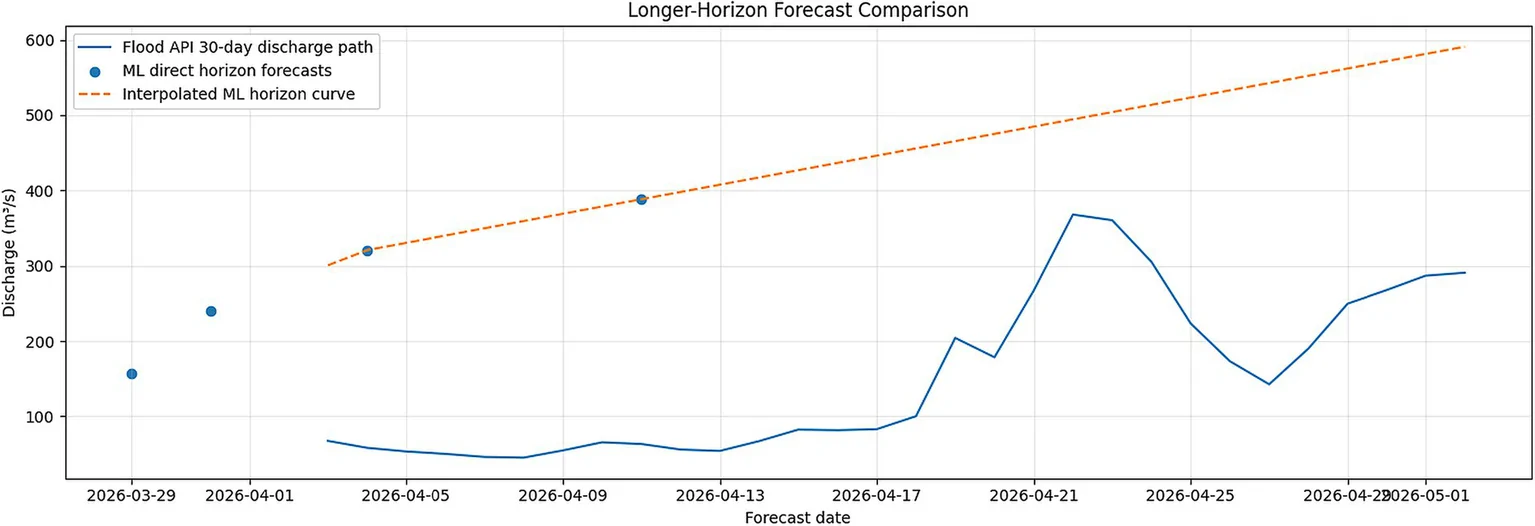
Single-issuance direct emulator points compared with the native 30-day Flood API trajectory. This is a consistency check, not a historical forecast-skill comparison.

The direct horizon points also differed from the native 30-day Flood API trajectory in absolute level and temporal shape. Because the Flood API already provides forecasts at the same lead times, the emulator must be compared against archived native forecasts before any added-value claim is possible. The present figure demonstrates disagreement only; it does not establish that the emulator is more local, more accurate, or more informative than GloFAS (see Figure 18).

### 4.6 Exploratory classification of GloFAS-derived high-flow product states

The binary target identified next-day GloFAS-derived values at or above the full-series 0.90 quantile. The holdout contained 16 positives (4.38%). XGBCls had the highest accuracy (0.962) and F1 (0.667), while ExtraTreesCls had recall 0.938 and PR-AUC 0.851. Because the cutoff was defined using the full series and the states are not observed floods, these metrics describe exploratory discrimination of a rare product state rather than operational event detection (see Table 6).

**Table 6:** Exploratory binary classification of the ≥0.90-quantile GloFAS-derived next-day state.

Model	Threshold used	Accuracy	Balanced Accuracy	Precision	Recall	F1	ROC-AUC	PR-AUC	Brier
ExtraTreesCls	0.235	0.945	0.942	0.441	0.938	0.600	0.984	0.851	0.0207
XGBCls	0.075	0.962	0.920	0.538	0.875	0.667	0.984	0.766	0.0208
HistGBC	0.050	0.942	0.880	0.419	0.813	0.553	0.981	0.761	0.0216
Logit	0.090	0.885	0.910	0.268	0.938	0.417	0.963	0.603	0.0397

Label cutoffs were calculated from the full series and therefore contain mild label-definition leakage.

Differences between XGBCls and ExtraTreesCls represent alternative error trade-offs within the product-state task. They cannot be translated into false-alarm or missed-flood costs because no observed flood labels or institutional thresholds were used.

The multicategory holdout comprised 328 Normal, 20 Watch, 14 Warning, and only 3 Extreme product states. ExtraTreesRegime achieved accuracy 0.923 but balanced accuracy 0.454 and Macro-F1 0.457, showing that the overall score was driven by the dominant Normal class (see Table 7).

**Table 7:** Exploratory classification of empirical GloFAS-derived product states.

Model	Accuracy	Balanced accuracy	Macro-F1	Weighted-F1
ExtraTreesRegime	0.923	0.454	0.457	0.920
HistGBRegime	0.915	0.377	0.381	0.904

Class names do not represent official flood-warning levels.

ExtraTreesRegime classified 322/328 Normal, 11/20 Watch, 4/14 Warning, and 0/3 Extreme cases correctly. The Extreme result is inconclusive rather than a stable estimate of zero detection: with only three cases, the exact 95% binomial interval for sensitivity extends from 0% to approximately 70.8%. The appropriate conclusion is that the sample is too small to estimate Extreme-state performance.

**Figure 19:**
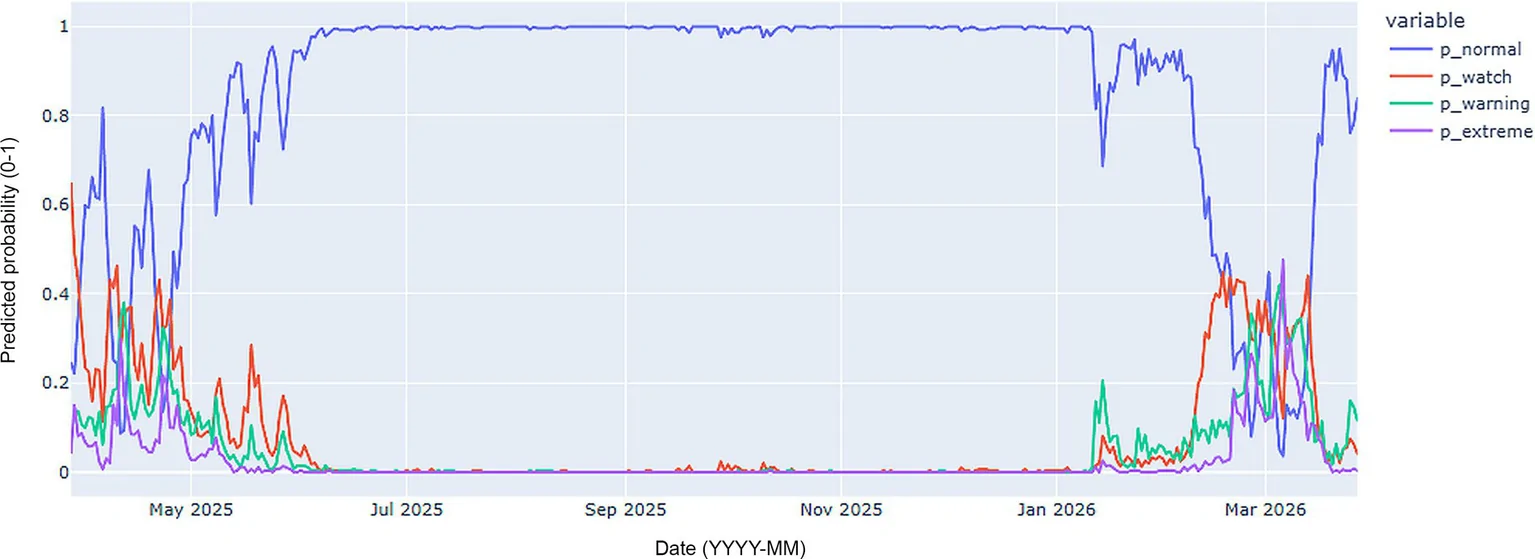
Estimated probabilities of empirical GloFAS-derived product states from ExtraTreesRegime. The curves are not official flood-warning probabilities.

Temporal class probabilities varied near the beginning and end of the holdout, but this apparent coherence remains descriptive because the classes are empirical quantiles of the GloFAS-derived target and the rarest class is inadequately represented (see Figure 19).

In summary, the retained evidence supports three bounded findings. First, the GloFAS-derived signal is highly emulatable at t+1 because current and recent source-product values dominate. Second, performance and interval calibration deteriorate with lead time, but the magnitude of deterioration is uncertain because backtesting used only three 2020 blocks and no repeated-run inference. Third, neither the prospective disagreement with the native Flood API nor the sparse classification results support operational or real-river claims.

## 5 Discussion

### 5.1 Interpretation of multi-horizon product emulation

The multi-horizon results show that the local model is most defensible as a short-term emulator of the GloFAS-derived product, not as an independently validated river forecast. In rolling-origin testing, ElasticNetCV achieved mean R^2^/NSE values of 0.777 at t+1 and 0.334 at t+3, but skill became negative at t+7 (−0.065) and t+14 (−0.609). The final 365-day holdout was more favorable (0.900, 0.687, 0.535, and 0.424, respectively), indicating that model performance depends materially on the evaluated hydrological regime and period. The improvement over one-step persistence at t+1 shows that the engineered feature set contains useful short-term structure beyond a naive carry-forward rule; however, the sharp difference between the 2020 backtest blocks and the 2025–2026 holdout prevents the final-holdout scores from being interpreted as stable operational skill. In time-series forecasting, such divergence is precisely why chronological validation across multiple origins and seasons is necessary (Bergmeir and Benítez, 2012). Therefore, the principal contribution is a horizon-specific diagnostic of product emulatability: t+1 is consistently the most credible horizon, t+3 is conditionally useful, and longer horizons require stronger validation and direct comparison with the native forecast.

The explainability results clarify why the short horizon performs well. Current GloFAS-derived discharge had a permutation importance of 0.6969 at t+1, far exceeding the next-ranked predictors, which confirms that the emulator primarily exploits persistence and recent state information in the source product. This is compatible with the behavior of a routed hydrological series, but it must not be interpreted as evidence that the model independently reconstructed local rainfall-runoff processes. GloFAS is produced through LISFLOOD, meteorological forcing, routing, calibration, and regionalisation that incorporate streamflow observations where available (Alfieri et al., 2013; Van der Knijff et al., 2010; Harrigan et al., 1979; Hirpa et al., 2018; Harrigan et al., 2023; Copernicus Emergency Management Service, 2025; Copernicus Emergency Management Service, 2024); consequently, the local model learns a signal that already contains substantial large-scale hydrological structure. At t+7 and t+14, the relative importance of temperature, wet-season indicators, calendar terms, and accumulated rainfall increased as direct autoregressive information decayed. That shift identifies where local meteorological and hydrometric information may add value, but the missing split-level weather-only and discharge-history-only ablations mean that incremental meteorological skill cannot yet be quantified. This distinction is especially important in Ecuador and the Andes, where gridded meteorological products exhibit spatially heterogeneous errors and often benefit from local adjustment (Magoffin et al., 2023; Iza-Wong et al., 2026; Fernández-Palomino et al., 2022; Condom et al., 2020; Muñoz-Sabater et al., 2021; Beck et al., 2016).

### 5.2 Benchmarking, uncertainty, and operational implications

The native Flood API forecast is the relevant operational benchmark because it already provides predictions at the same lead times (Harrigan et al., 2023; Open-Meteo, 2026). In the single-issuance comparison, the recursive emulator exceeded the native trajectory by approximately 160 m^3^/s on average and drifted upward while the native path remained much lower. Without observed discharge or archived native forecasts for matching historical issuance dates, this disagreement cannot determine which trajectory is closer to the physical river; it instead exposes a consistency and model-governance problem. A local emulator should be considered for use only when it demonstrates repeated improvement over archived native forecasts under the same origins, horizons, and target dates. Otherwise, the native GloFAS product should remain the primary reference and the emulator should be limited to explanation, sensitivity analysis, or local scenario exploration. This decision rule avoids presenting two unranked forecasts as equivalent and gives the prospective comparison a clear practical meaning. It also shows why recursive deployment is riskier than direct horizon-specific prediction: small one-step errors can accumulate and move the simulated path away from the product family used to train the model.

The uncertainty analysis reveals a calibration problem that becomes more serious with lead time. Empirical coverage of the nominal 80% interval declined from 74.5% at t+1 to 59.7% at t+7 and 54.8% at t+14. At the same time, average width increased from 81.221 m^3^/s at t+1 to 164.952 m^3^/s at t+7, but then narrowed to 135.189 m^3^/s at t+14. The combination of lower coverage and a narrower longest-horizon interval indicates under-dispersion or mis-centring rather than a simple increase in uncertainty. Independent quantile models can also generate crossing or internally inconsistent intervals when no non-crossing constraint or rearrangement is applied (Koenker and Bassett, 1978; Chernozhukov et al., 2010). The residual pattern supports the same caution: errors were approximately centred at t+1 but became more dispersed at higher predicted values, showing that the model is least reliable during the high-value states that are most consequential for flood-related interpretation. A stronger probabilistic workflow should estimate all horizons, enforce coherent quantiles, evaluate calibration within each rolling split and hydrological season, and report both coverage and sharpness rather than relying on nominal interval levels alone.

The classification modules should remain explicitly exploratory. The binary and multicategory labels were defined from full-series GloFAS quantiles, which introduces label-definition leakage and does not correspond to observed floods or official warning thresholds. Moreover, the holdout contained only 16 binary positives and three Extreme cases; therefore, high overall accuracy is dominated by the majority class, while sensitivity for the rarest state is too uncertain for operational interpretation. The current results are useful for demonstrating class imbalance and alternative error trade-offs, but they cannot support claims about missed floods, false alarms, or warning-system performance. A defensible extension would derive all thresholds within each training window, retain those thresholds unchanged in validation and testing, evaluate precision-recall measures and class-specific uncertainty across multiple seasons, and link product states to independent hydrometric or civil-protection evidence before assigning operational labels.

### 5.3 Limitations, reproducibility, and research agenda

Several limitations define the boundaries of the study. First, the target is a GloFAS-derived grid-cell series near Babahoyo, and the returned cell has not yet been verified against a named river reach, drainage area, or channel identifier. Second, continuous local gauge discharge was unavailable, so the study evaluates agreement with the source product rather than accuracy for the Babahoyo River. The INAMHI yearbooks strengthen geographic and seasonal context, but they report historical water levels and cannot be compared directly with discharge without a rating curve. Third, temporal validation used only three 90-day blocks within January–September 2020, one final holdout, one random seed, and fixed model configurations; model rankings are therefore descriptive and may be sensitive to regime, tuning, and sampling. Fourth, the archived materials do not yet contain complete split-level ablation results, repeated-run dispersion, the t+3 prediction interval, or historical native-forecast archives. Finally, API-based products may evolve through changes in forcing, calibration, processing, or versioning, so frozen responses and exact query metadata are necessary for reproducibility.

These limitations also define a concrete research agenda. The immediate priority is to complete the INAMHI yearbook digitisation and manual verification, screen candidate GloFAS cells using independent historical timing and seasonality, and document the selected cell’s hydrological correspondence. The forecasting analysis should then be rerun with rolling origins distributed across wet and dry seasons, training-only label thresholds, repeated seeds, nested or equivalently constrained model tuning, complete feature-family ablations, coherent intervals at all four horizons, and matched archived native GloFAS forecasts. Where accessible, observed gauge discharge and rating-curve information should be incorporated as the decisive validation layer. Replication across multiple INAMHI stations and GloFAS cells would determine whether the horizon-dependent pattern is site-specific or transferable. A versioned repository containing frozen API responses, extracted yearbook tables, dependency versions, split-level metrics, figures, and model artifacts would align the workflow with FAIR reproducibility principles (Wilkinson et al., 2016). Under this expanded design, the current single-site case can serve as a transparent methodological prototype rather than as evidence of universal operational performance.

## 6 Conclusion

This study developed a transparent local post-processing framework for a GloFAS v4-derived discharge product near Babahoyo and connected it with an explicit historical hydrometric-context protocol based on official INAMHI yearbooks. The strongest finding was the high t+1 agreement and clear improvement over persistence, supported by interpretable dependence on current and recent source-product states. t+3 retained positive average skill, while t+7 and t+14 identified the point at which performance becomes more regime-sensitive and broader information is required.

The resulting contribution is a horizon-aware decision framework rather than a universal replacement for GloFAS. It shows when a lightweight local emulator is analytically useful, how uncertainty and predictor dependence change with lead time, and how historical INAMHI levels can be linked to matching Flood API dates for cell screening and event-timing corroboration. The next empirical step is to complete the yearbook-table digitisation, archive matched native reforecasts, regenerate split-level ablations across more seasons, and apply the protocol to additional stations. These extensions build directly on the demonstrated short-term skill and make the design suitable for broader evaluation in data-scarce tropical settings.

## Data Availability

Publicly available datasets were analyzed in this study. This data can be found here: https://github.com/aharo8014/Explainable-Machine-Learning-for-Multi-Horizon-River-Dis-charge-Forecasting-Prediction-Intervals.

## References

[ref1] Alfieri L. Burek P. Dutra E. Krzeminski B. Muraro D. Thielen J. . (2013). GloFAS—global ensemble streamflow forecasting and flood early warning. Hydrol. Earth Syst. Sci. 17, 1161–1175. doi: 10.5194/hess-17-1161-2013

[ref2] Beck H. E. van Dijk A. I. J. M. de Roo A. Miralles D. G. McVicar T. R. Schellekens J. . (2016). Global-scale regionalization of hydrologic model parameters. Water Resour. Res. 52, 3599–3622. doi: 10.1002/2015WR018247

[ref3] Bergmeir C. Benítez J. M. (2012). On the use of cross-validation for time series predictor evaluation. Inf. Sci. 191, 192–213. doi: 10.1016/j.ins.2011.12.028

[ref4] Chernozhukov V. Fernández-Val I. Galichon A. (2010). Quantile and probability curves without crossing. Econometrica 78, 1093–1125. doi: 10.3982/ECTA7880, 42550984

[ref5] Condom T. Martínez R. Pabón J. D. Costa F. Pineda L. Nieto J. J. . (2020). Climatological and hydrological observations for the south American Andes: in situ stations, satellite, and reanalysis data sets. Front. Earth Sci. 8:92. doi: 10.3389/feart.2020.00092

[ref6] Copernicus Emergency Management Service. (2024). GloFAS v4 Calibration Methodology and Parameters Reading European Centre for Medium-Range Weather Forecasts. (Accessed July 19, 2026). Available online at: https://confluence.ecmwf.int/spaces/CEMS/pages/340755426/GloFAS+v4+calibration+methodology+and+parameters

[ref7] Copernicus Emergency Management Service. (2025). GloFAS v4.0 Reading European Centre for Medium-Range Weather Forecasts. (Accessed July 19, 2026). Available online at: https://confluence.ecmwf.int/spaces/CEMS/pages/388505179/GloFAS+v4.0

[ref8] Diebold F. X. Mariano R. S. (1995). Comparing predictive accuracy. J. Bus. Econ. Stat. 13, 253–263. doi: 10.1080/07350015.1995.10524599

[ref9] Fernández-Palomino C. A. Hattermann F. F. Krysanova V. Lobanova A. Vega-Jácome F. Lavado W. . (2022). A novel high-resolution gridded precipitation dataset for Peruvian and Ecuadorian watersheds: development and hydrological evaluation. J. Hydrometeorol. 23, 309–336. doi: 10.1175/JHM-D-20-0285.1

[ref10] Harrigan S. Zsoter E. Alfieri L. Prudhomme C. Salamon P. Wetterhall F. . (1979). GloFAS-ERA5 operational global river discharge reanalysis 1979-present. Earth Syst. Sci. Data 12, 2043–2060. doi: 10.5194/essd-12-2043-2020

[ref11] Harrigan S. Zsoter E. Cloke H. Salamon P. Prudhomme C. (2023). Daily ensemble river discharge reforecasts and real-time forecasts from the operational global flood awareness system. Hydrol. Earth Syst. Sci. 27, 1–19. doi: 10.5194/hess-27-1-2023

[ref12] Hirpa F. A. Salamon P. Beck H. E. Lorini V. Alfieri L. Zsoter E. . (2018). Calibration of the global flood awareness system (GloFAS) using daily streamflow data. J. Hydrol. 566, 595–606. doi: 10.1016/j.jhydrol.2018.09.052, 32226131 PMC7097972

[ref13] Hrachowitz M. Savenije H. H. G. Blöschl G. McDonnell J. J. Sivapalan M. Pomeroy J. W. . (2013). A decade of predictions in ungauged basins (PUB)—a review. Hydrol. Sci. J. 58, 1198–1255. doi: 10.1080/02626667.2013.803183

[ref14] Instituto Nacional de Meteorología e Hidrología. (1990). Anuario Hidrológico 1990. Quito INAMHI. (Accessed July 19, 2026). Available online at: https://www.inamhi.gob.ec/biblioteca/

[ref15] Instituto Nacional de Meteorología e Hidrología. (1991). Anuario Hidrológico 1991. Quito INAMHI. (Accessed July 19, 2026). Available online at: https://www.inamhi.gob.ec/biblioteca/

[ref16] Instituto Nacional de Meteorología e Hidrología. (1992). Anuario Hidrológico 1992. Quito INAMHI. (Accessed July 19, 2026). Available online at: https://www.inamhi.gob.ec/biblioteca/

[ref17] Instituto Nacional de Meteorología e Hidrología. (1993). Anuario Hidrológico 1993. Quito INAMHI. (Accessed July 19, 2026). Available online at: https://www.inamhi.gob.ec/biblioteca/

[ref18] Instituto Nacional de Meteorología e Hidrología. (2026). Publicaciones de la Dirección de la Información Hidrometeorológica. Quito: INAMHI; (Accessed July 19, 2026). Available online at: https://www.inamhi.gob.ec/publicaciones-de-la-direccion-de-la-informacion-hidrometeorologica/

[ref19] Iza-Wong A. Moldovan G. Ben Bouallègue Z. Hemingway R. Chantry M. Lavers D. A. (2026). Evaluation of precipitation observations across Ecuador. Atmos. Sci. Lett. 27:e70002. doi: 10.1002/asl.70002

[ref20] Knoben W. J. M. Freer J. E. Woods R. A. (2019). Technical note: inherent benchmark or not? Comparing Nash-Sutcliffe and Kling-Gupta efficiency scores. Hydrol. Earth Syst. Sci. 23, 4323–4331. doi: 10.5194/hess-23-4323-2019

[ref21] Koenker R. Bassett G. Jr. (1978). Regression quantiles. Econometrica 46, 33–50. doi: 10.2307/1913643

[ref22] Kratzert F. Klotz D. Shalev G. Klambauer G. Hochreiter S. Nearing G. (2019). Towards learning universal, regional, and local hydrological behaviors via machine learning applied to large-sample datasets. Hydrol. Earth Syst. Sci. 23, 5089–5110. doi: 10.5194/hess-23-5089-2019

[ref23] Magoffin R. H. Hales R. C. Erazo B. Nelson E. J. Larco K. Miskin T. J. (2023). Evaluating the performance of satellite derived temperature and precipitation datasets in Ecuador. Remote Sens. 15:5713. doi: 10.3390/rs15245713

[ref24] Muñoz-Sabater J. Dutra E. Agustí-Panareda A. Albergel C. Arduini G. Balsamo G. . (2021). ERA5-land: a state-of-the-art global reanalysis dataset for land applications. Earth Syst. Sci. Data 13, 4349–4383. doi: 10.5194/essd-13-4349-2021

[ref25] Nash J. E. Sutcliffe J. V. (1970). River flow forecasting through conceptual models part I—a discussion of principles. J. Hydrol. 10, 282–290. doi: 10.1016/0022-1694(70)90255-6

[ref26] Open-Meteo. (2026). Global Flood API Documentation. (Accessed July 19, 2026). Available online at: https://open-meteo.com/en/docs/flood-api

[ref27] Razavi S. Tolson B. A. Burn D. H. (2012). Review of surrogate modeling in water resources. Water Resour. Res. 48:W07401. doi: 10.1029/2011WR011527

[ref28] Van der Knijff J. M. Younis J. De Roo A. P. J. (2010). LISFLOOD: a GIS-based distributed model for river basin scale water balance and flood simulation. Int. J. Geogr. Inf. Sci. 24, 189–212. doi: 10.1080/13658810802549154

[ref29] Wilkinson M. D. Dumontier M. Aalbersberg I. J. Appleton G. Axton M. Baak A. . (2016). The FAIR guiding principles for scientific data management and stewardship. Sci. Data 3:160018. doi: 10.1038/sdata.2016.18, 26978244 PMC4792175

